# Low-Contrast Thermal Resolution Test Targets: A New Approach

**DOI:** 10.6028/jres.095.051

**Published:** 1990

**Authors:** John Geist, Donald B. Novotny

**Affiliations:** National Institute of Standards and Technology, Gaithersburg, MD 20899

**Keywords:** low-contrast thermal resolution, radiometry, target fabrication, thermal contrast modeling, thermal radiation, thermal resolution target

## Abstract

A new type of thermal resolution test target optimized to minimize the effects of lateral thermal gradients at low thermal contrast is described. This target consists of thin-film inconel heater strips over an etched silica substrate bonded to an aluminum heat sink. A simple, finite-difference model is used to study how variations in target construction and materials affect the generated thermal resolution test pattern. The construction, testing, and use of this type of target to extend the lower end of the contrast range of a conventional target are described.

## 1. Introduction

Thermal resolution test targets are used to characterize the performance of infrared imaging systems [1]. Low-contrast test targets are used to measure the minimum resolvable temperature difference [2] that can be distinguished by these systems.

**Figure 1:**
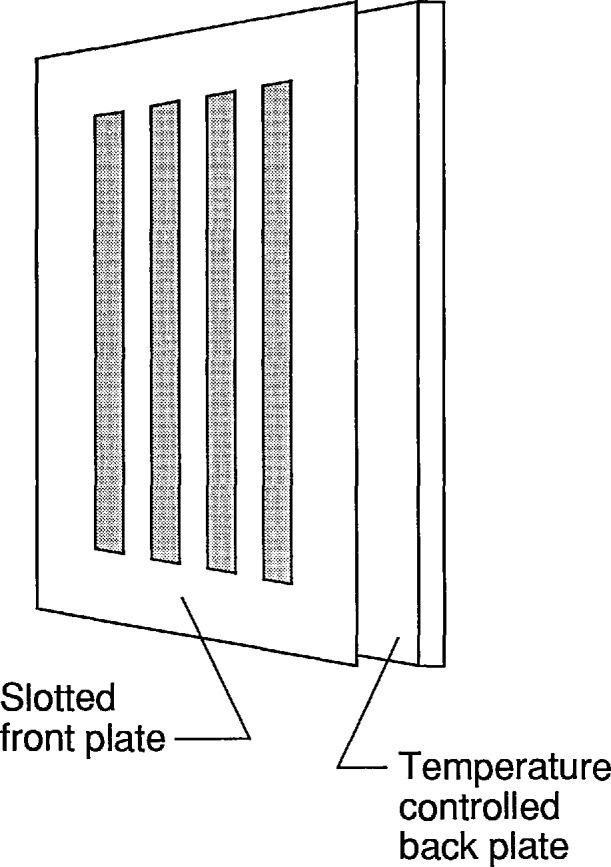
A conventional thermal resolution test target. The temperature of the back plate is controlled to a constant temperature difference relative to the front plate. The temperature of the latter is allowed to float.

A commonly used thermal resolution test target of conventional design is shown in figure 1. The front plate is blackened so that it is an efficient emitter of infrared radiation. The back plate, which is also blackened, is visible through the slots in the front plate. The temperature of the front plate is allowed to float with the ambient, and the temperature difference between the back and front plates is sensed by a thermocouple. An electrical current flowing through a heater covering the rear surface of the back plate is controlled to maintain the temperature difference between the back and front plates at a user-adjustable value.

The simple design of figure 1 works very well for temperature differences of the order of 10 K and has been used successfully for much smaller temperature differences. Unfortunately, an air gap exists between the front and back plate causing a lack of tight coupling between the temperature distribution over the back plate and that of the front plate. As a result, it is possible to have small lateral temperature gradients across the back plate that are not correlated with similar gradients in the front plate. The temperature difference at any slot is the sum of the intentional temperature difference set by the user and the difference between the uncontrollable temperature gradients. This creates a problem at low contrasts.

As the temperature difference between the two plates is reduced, these uncorrelated gradients begin to interfere with the pattern generated by the slots in the front plate. For very small temperature differences between the plates, the uncorrelated lateral thermal gradients can result in the slots appearing hotter than the background on one side of the target, while appearing cooler than the background on the other side. Consequently, it is not possible to know the actual value of the thermal contrast at any slot in such a situation.

During the last 20 years, the performance of thermal imagers has improved so much that it will soon be necessary to test systems with thermal contrasts of the order of 1 mK. It is not at all clear that the conventional design can meet this need. The purpose of this paper is to describe a new design specifically tailored for low thermal contrast. The principal advantage of this new design is that the lateral temperature gradients in the hotter surface are strongly correlated with those in the cooler surface as a result of the way the thermal contrast is generated.

**Figure 2:**
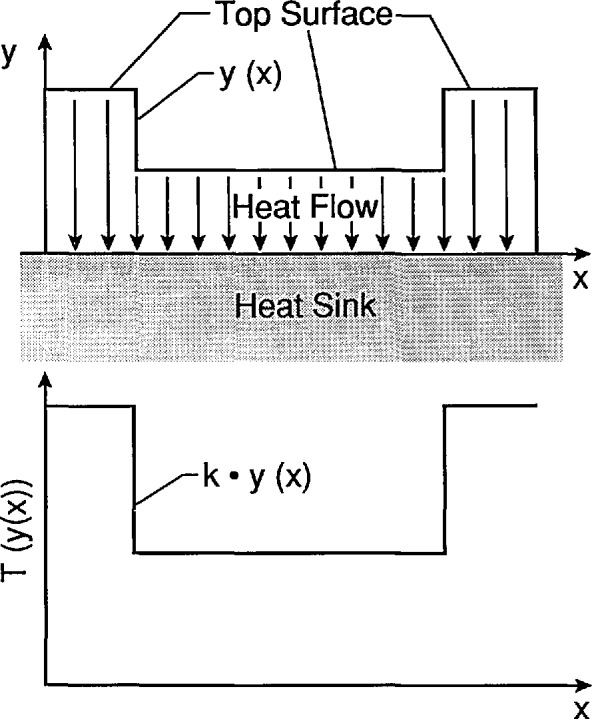
Cross section of a target of the new design illustrating the principle for achieving low thermal contrast with minimum influence from parasitic temperature gradients. Power is dissipated with uniform density over the top surface of a low thermal conductivity material whose bottom surface is in good thermal contact with a heat sink. The only path for heat flow is through the low thermal conductivity material. The function *y*(*x*) is the height of the top surface of that material, and the temperature profile at that surface is proportional to *y*(*x*).

A cross section of the new design is illustrated in figure 2. The target consists of an insulating material whose bottom surface is in good thermal contact with a heat sink, and whose other surfaces are well insulated. Power is applied with a uniform density over the top surface of the material. The heat is confined to flow in the vertical direction by the boundary conditions and the geometry of the device. In this case, the temperature profile over the top surface will follow its contour as illustrated in figure 2 and discussed in more detail in the next section of this paper.

**Figure 3:**
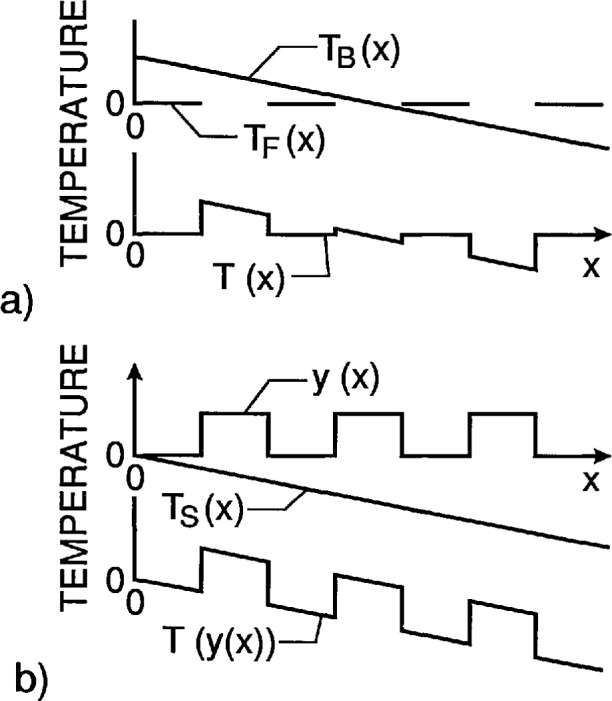
Comparison of effect of temperature gradients on new and conventional approaches to thermal resolution targets. Part a) applies to a conventional target. *T*_F_(*x*) is the temperature of the front plate between the slots, *T*_B_(*x*) is the temperature of the back plate, and *T*(*x*) is the temperature profile generated by the target. Part b) applies to a target based on the design described in this paper. The function *y*(*x*) is the height profile of the top surface of the target, *T*_s_(*x*) is the temperature gradient in the target substrate, and *T*[*y*(*x*)] is the temperature profile generated by the target.

Figure 3 illustrates the different effects of lateral thermal gradients on the thermal contrast displayed by the conventional design and the new design. For this comparison, it is assumed that 1) the same lateral gradient exists in the temperature *T*_B_(*x*) of the back plate of the conventional target and in the temperature *T*_s_(*x*) of the substrate of the new type target, 2) the temperature of the back plate of the conventional target is controlled to exceed the temperature of the front plate by a fixed difference at the point *x* =0, and 3) the power dissipated in the new type target is set to give that same temperature difference at the points of discontinuity in the height profile *y*(*x*). The striking feature is that the distortion of the temperature profile *T*(*x*) generated by the conventional target deviates from the ideal much more than that of the temperature profile *T*[*y*(*x*)] generated by the new type of target.

The next section of this paper presents the theory of operation of a thermal test target based on the new design. That section is followed by one that describes some targets built to demonstrate the new design, a section that compares the measured performance of these devices with the theoretical predictions, and a final section that illustrates how the targets would be used to extend the contrast range of a conventional target.

## 2. Theory of Operation

### 2.1 The Model

**Figure 4:**
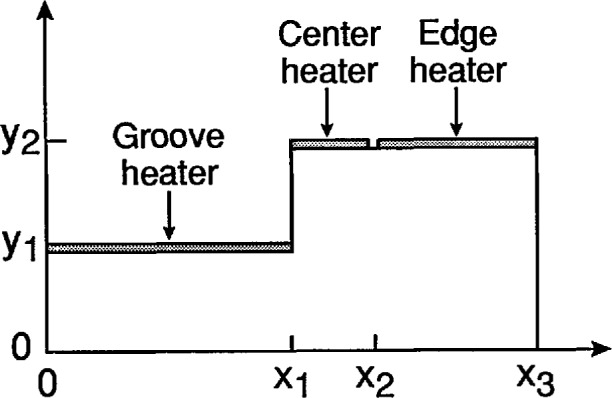
An idealized device for computer modeling of various sources of error affecting the new type of thermal resolution target described in this paper.

**Figure 5:**
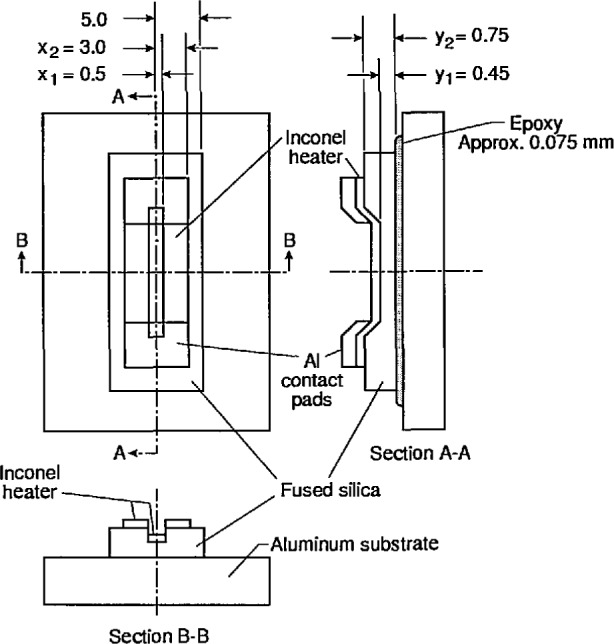
Schematic diagram of the fabricated target (not drawn to scale). All dimensions are in millimeters. The views A-A and B-B are cross sections of thin slices taken at the positions indicated. The heights of these cross-sectional views are exaggerated to illustrate the inconel and aluminum films and the shape of the groove. The X and Y labels correspond to those in figure 4.

An idealized device is used to model the heat transfer and temperature distribution; its cross section simulates a thin slice of material of the actual device which is shown in view B-B of figure 5. The symmetry properties of this device were used extensively in this model. Figure 5 shows the mirror symmetry about each center line. Consequently, figure 4 extends only from the center line to the edge along section-line B-B. Because mirror symmetry exists about the center line B-B, a temperature profile for this cross section is obtained using a two-dimensional model.

The following assumptions were made for this model:

1. The bottom surface of the device is in contact with a perfect heat sink, and all temperatures are measured relative to the temperature of the heat sink;
2. The top surface of the device has three heaters, a groove heater, a center heater, and an edge heater, and they can be independently heated;
3. The sides and the top surfaces above the three heaters are composed of perfect insulators;
4. The heat transfer from the top surface of the device to the heat sink is entirely by conduction through the device; and
5. Steady-state conditions exist; the temperature is not a function of time.

The model is a finite difference model and consists of the cross-sectional area of figure 4 being divided into cells. A sketch of this cell construction is shown in Appendix 1. All *x*-cell dimensions (the lengths of the cell boundaries) are equal, and all *y*-cell dimensions (the heights of the cell boundaries) are equal. The temperature of each cell is that at its center. The heat flow equations are written in integral form for each family of cells described below:

#### Interior cells

The cell dimensions are *x* by *y.* The net flow of heat into the cell is zero which means that the total heat flowing into the cell is equal to the total heat flowing out of the cell. As an example, the equation for the net heat flow into this cell per unit length, *F*(*m, n*), is given by

$$\begin{array}{l} 0=F\left(m,n\right)=k\{\left(x/y\right)[\left(T\left[m,\;n+1\right]-T\left[m,\;\underline{n}\right]\right)\\ \;+\left(T\left[m,n-1\right]-T\left[m,n\right]\right)]+\left(y/x\right)[(T[m\\ \;-1,n]-T\left[m,n\right])+(T\left[m+1,n\right]\\ \;-T\left[m,n\right])]\}, \end{array}$$

where:

*T*[*m*, *n* ] is the temperature of the *m*, *n* interior cell,
*k* is the thermal conductivity of the material in watts per unit length per degree kelvin,
*x* or *y* in the numerator is the length of the cell face across which heat flows, and
*x* or *y* in the denominator is the distance between the centers of two adjacent cells.

#### Surface cells with heaters

The cell dimensions are *x* by *y*/2. The heat flow into this cell from the heater, *F*(*m*, *n*), is equal to the heat flow across the lower horizontal cell boundary plus the net heat flow across the two vertical cell boundaries. Note that here *F*(*m, n*) is the heat per unit length supplied by the heater and not the net flow into the cell and is not zero.

#### Exterior corner cell such as the cell of point (x_1_, y_2_) in figure 4

The cell dimensions are *x/*2 by *y/*2. The heater on the horizontal surface of this cell is half the size of those on the surface cells; consequently, the heat flow from the heater is only half that from the surface cell heater. This heat flow into the cell is equal to the heat flow across the lower boundary of the cell plus the heat flow across the vertical boundary of the cell. Because the heater and cell boundaries are both one-half of the dimensions of those for the interior cell, the heat flow equation obtained is the same as that for an interior cell. Again, *F*(*m, n*) is the heat per unit length supplied by the heater and not the net flow into the cell and is not zero.

#### Interior corner cell such as the cell of point (x_1_, y_1_) of figure 4

The cell dimensions going clockwise around the cell, starting with the heater dimension are *x/*2 by *y/*2 by *x/*2 by *y* by *x* by *y/*2. The heat flowing into this cell from the heater, which is half the heat flowing into a surface cell, is equal to the net heat flowing across the vertical boundaries plus the net heat flowing across the horizontal boundaries. The heat flowing across the surface represented by the first *y/*2 dimension of this cell is zero.

Boundaries across which no heat flows because of the constraints imposed by this model (assumption 3 above when no heater is present on the surface) are handled by setting the average temperature gradient across this surface equal to zero. This is done, for the algorithm given in the program in Appendix 1, by setting up virtual cells adjacent to the surface cells and then setting their temperatures the same as those of the adjacent (now interior) surface cells.

Surface cells with heater elements are handled by this algorithm by setting up virtual cells as before. The heat dissipated by the heaters is doubled by the program to accommodate the imaginary heat flow into the virtual cells and thereby provide the specified heat flow into the surface cells under the heaters.

The equations obtained by applying all of the cell conditions stated are solved for the temperature of the cell. The program source code is listed in Appendix 1 and can be compiled by version 3 of TURBO PASCAL.^1^,^2^ The program first sets all the temperatures to zero, the temperature of the heat sink. It then applies the heat dissipation specified for the heaters and successively calculates the temperatures of all cells. This process is repeated 500 times. Every successive iteration uses the temperatures calculated in the previous iteration with the same specified heater power dissipation. The temperatures of all the cells converge to within a small fraction of 1% of their final values in this many iterations.

**Figure 6:**
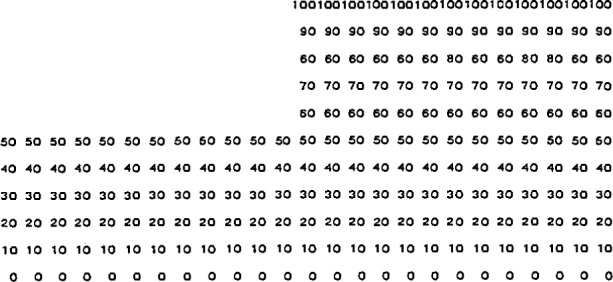
A typical result of the computer program listed in Appendix 1 for the device of figure 4 when the groove and edge heaters are dissipating the same power density as the central heater. For this example, there are 25 nodes on the *x*-axis, 11 nodes on the *y*-axis, *y*_1_ = 0.5 mm, *y*_2_ = 1.0 mm, *x*_1_ = 2.5 mm, *x*_2_ = 3.33 mm, and *x*_3_ = 5 mm, the lower left node is at the point (0 mm, 0 mm), and the upper right node is at the point (5 mm, 1 mm).

This program also allows the width and the depth of the groove to be independently varied in discrete steps, the power density in the edge heater to be set to zero or to the same value as that in the center heater, and the power density in the groove heater to be set to be a fraction between zero and the value set for the center heater. A typical result with the power density in the groove and edge heaters set the same as that in the center heater is shown in figure 6. The power density per unit conductivity in the center heater was adjusted to normalize the temperature at the top surface to 100 for these calculations. The same value was used in later calculations having the same geometry.

The important point of figure 6 is that the temperature profile of the top surface follows the physical profile of the top surface. This result is a direct consequence of the assumptions used in deriving the model, and of the assumption of uniform dissipation of the same power density in all heaters. How well these assumptions can be realized in practice will, in part, determine how well the performance of a real device can approach the performance of the idealized device. The next three figures show some of the deviations from idealized performance that can be expected with different power densities in the device heaters.

**Figure 7:**
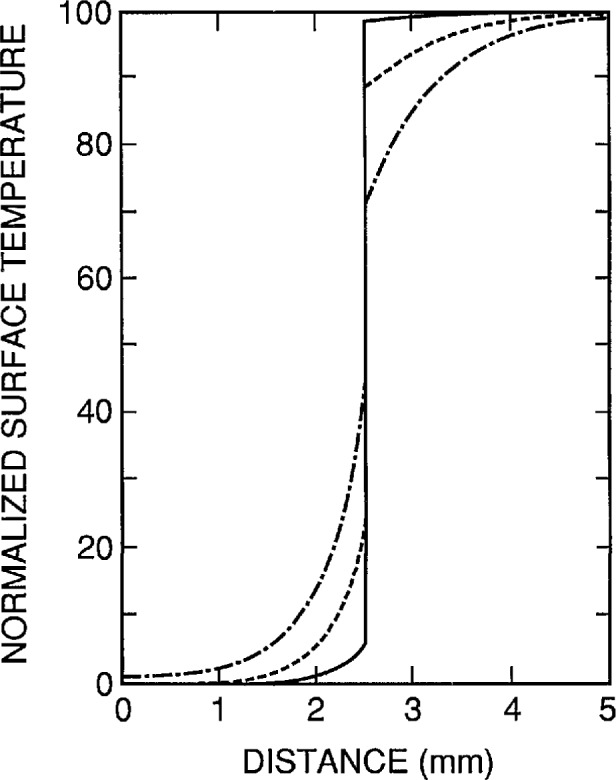
Normalized surface temperature for the device of figure 4 when the edge and center heaters are dissipating the same power density, but the groove heater is dissipating no power. The dimensions are the same as those of the device modeled in figure 6 except that the cases of *y*_1_=0.2 mm (solid line), *y*_1_ = 0.5 mm (dashed line), and *y*_1_ = 0.8 mm (dot-dash line) are considered.

**Figure 8:**
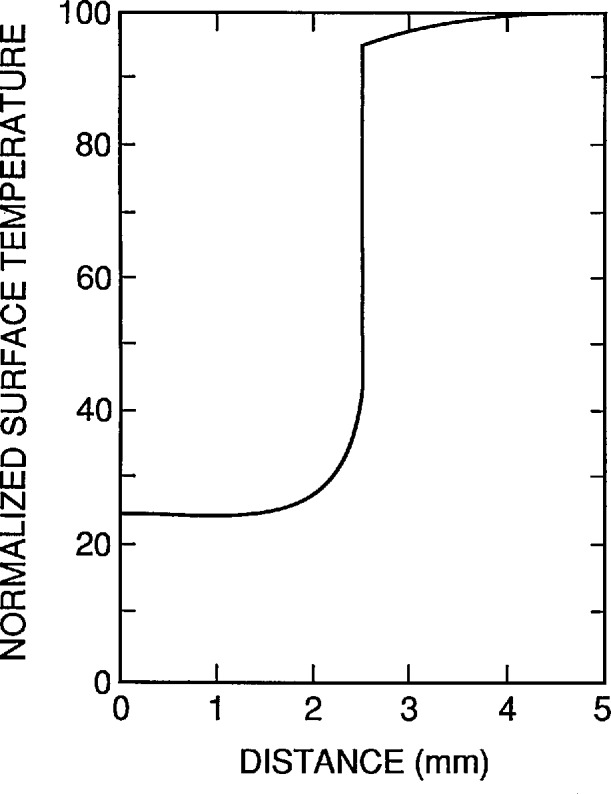
Normalized surface temperature for the device of figure 4 with the dimensions of the device modeled in figure 6 when the groove heater is dissipating half the power density being dissipated by the center and edge heaters.

Figure 7 shows the effect of groove depth on the temperature profile (with the same power density dissipated in the center and edge heaters and no power dissipated in the groove heater) for a target having the geometry shown in figure 4 and the same groove width and overall width as the target analyzed in figure 6. This figure shows that the temperature profile no longer follows the physical profile of the top surface of the target, and that the largest deviation from uniformity on each side of the temperature discontinuity occurs with shallow grooves. Therefore, for sharp thermal contrast, the groove heater must dissipate about the same power density as the center heater, and the target must have grooves that extend almost to the bottom of the low thermal conductivity material. The applications of these criteria are depicted in figure 8 which shows that fairly good uniformity on each side of the temperature discontinuity can be achieved with a groove depth that is half of the thickness of the low conductivity material and a power density in the groove that is half of that in the center heater.

**Figure 9:**
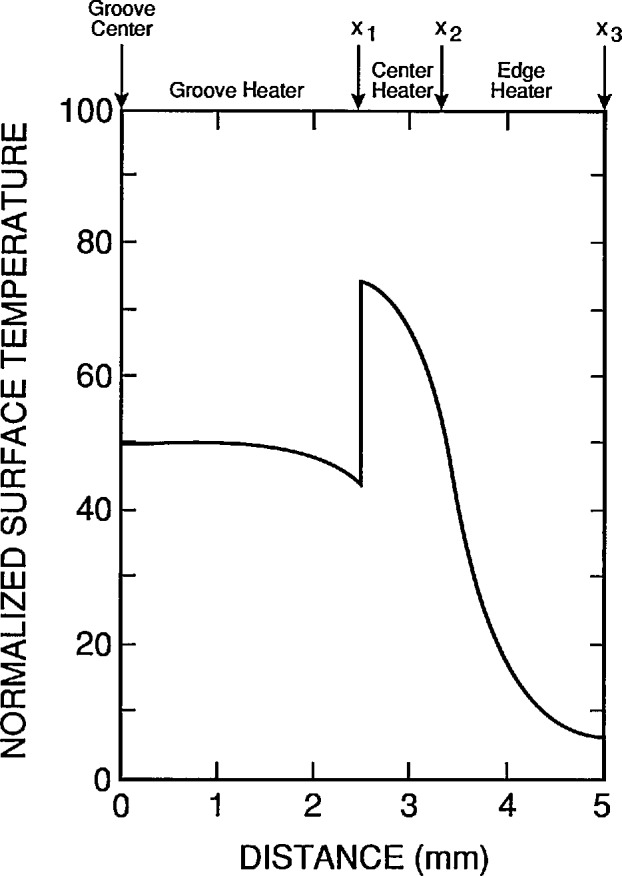
Normalized surface temperature for the device of figure 4 with the dimensions of the device modeled in figure 6 when the groove and center heaters are dissipating the same uniform power density, but the edge heater is off.

Figure 9 shows the effect of dissipating no heat in the edge heater while maintaining the same power density in the groove and center heaters. The loss of uniformity is striking, but a sharp discontinuity is still evident, even though the magnitude of the discontinuity is only 60% of its value in figure 6. The poor uniformity shows that, with no edge heater, the center heater should cover the entire top surface of the low conductivity material in a target of this type.

In the next section, devices are described that were built to test the criteria developed in this section.

## 3. Device Fabrication and Testing

A top view and two cross sections of one of the targets fabricated to test these criteria are shown in figure 5. In this target, there are no edge heaters, and the groove and center heaters are connected to the same aluminum contact pads.

**Figure 10:**
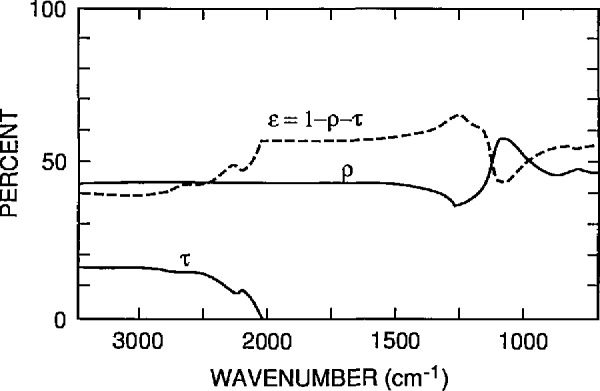
The emissivity *e* (dashed line) of the inconel-fused silica structure used in fabricating the devices of figure 5. The emissivity was calculated from *e* = 1 − *r* − *t*, from measured data for the reflectance *r* and the transmittance *t* of the structure. These data are also shown in the figure.

Fused silica (amorphous SiO_2_) was used as the low conductivity material because its thermal conductivity is low compared to that of aluminum, but high compared to that of air. A 10-nm-thick layer of sputter-deposited inconel was used as the surface heater because inconel has a relatively high resistivity, and this thickness of inconel film produced a heater with both a usable resistance between 100 and 250 Ω and a reasonably high infrared emissivity. Figure 10 shows the spectral dependence of the measured reflectance and transmittance, and the calculated emissivity, of a nominal 10-nm film of inconel on a 750-*µ*m- (0.030-in-) thick piece of fused silica. Aluminum was used for the heater contact pads. The fused silica device was attached to the heat sink with a fast-curing epoxy adhesive. Aluminum was used as the heat sink material because it is an inexpensive material that is easily machined and has a high thermal conductivity.

Table 1 compares the thermal conductivities of aluminum, fused silica, and air. Table 2 compares the electrical resistance per square of a 10-nm film of inconel and a 250-nm film of aluminum. Finally, notice that the net power density per unit temperature difference radiated by a black-body at 300 K is only about 0.5% of that conducted through a 1-mm thick piece of fused silica. All of these results suggest that the assumptions stated in connection with figure 5 are well approximated by this device.

The fused silica was cut and polished using conventional optical shop techniques into 75-mm- (3-in-) diameter, 750-*µ*m- (0.030-in-) thick wafers for subsequent processing. Rectangular grooves whose long axes were parallel to the future current directions were ultrasonically machined into the top surface of the fused silica wafers. The walls on the long axis of the groove were vertical to produce an abrupt change in temperature across them (as shown in figs. 4 and 5); the walls at the ends of the grooves were sloped to allow continuous metal film coverage permitting the current to go down into and up out of the groove.

Photolithographic techniques were used to produce the thin film heater and contact pads on the top surface of the fused silica. A number of different variations of the basic technique were tried in an attempt to find a technique that would allow the devices to be made without any special post-processing steps. Some approaches were found to be better than others, but no completely satisfactory approach was found. The presence of the grooves prevented the photoresist from behaving normally during spinning, curing, and etching. The approaches tried and the results obtained are described in Appendix 2.

After processing, the devices were cut from the wafers using standard techniques for fused silica. (Attempts to cut the wafers with a saw designed for dicing silicon wafers resulted in a great deal of wafer cracking, and breakage further limited the number of devices for testing. No such problems were encountered with the standard techniques for fused silica.) After cutting, the devices were mounted with a thin film of epoxy on 5-cm by 5-cm by 3-mm (2-in by 2-in by 1/8-in) heat sinks machined from soft (for higher conductivity) aluminum.

Two bare copper wires were used as heater leads. Two layers of epoxy were used to attach each wire near one end of the heat sink to provide strain relief while insulating the wire from the heat sink. Then one end of each wire was attached to a device contact with silver-filled lacquer. These fabricated devices were then tested for electrical continuity.

## 4. Device Performance

A PtSi camera operating in the 3- to 5-*µ*m thermal infrared was used to study the performance of one of the low-contrast thermal resolution targets described above. The output of the camera was available on a television monitor as a thermal image and on an oscilloscope as a voltage vs time graph of one of the individual scan lines making up the television image.

**Figure 11:**
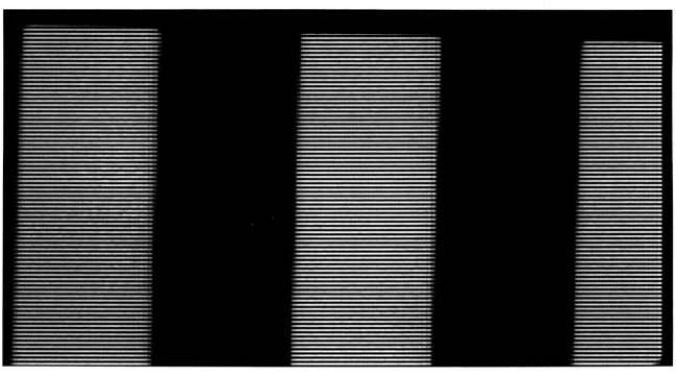
Television image of a portion of the conventional four-bar target used for calibrating the gain of the PtSi CCD-array camera. The bright lines are the television-scan lines. The thermal contrast between the bars and the background was 4 K, and the background was subtracted to give the dark field background shown.

**Figure 12:**
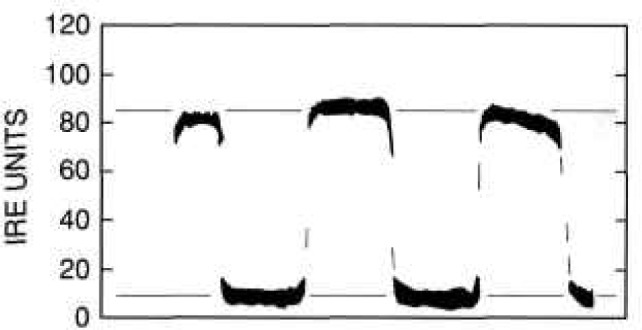
A scope trace of one of the scan lines in the image shown in figure 11. Nominal averages of the high- and low-temperature portions of the target are shown by the broken lines. The difference between the lines is about 75 IRE units.

First, the camera was focused on a portion of a conventional 10-cm-square (4-in-square), four-bar thermal resolution target. The thermal contrast between the rear heater and the slotted sheet was adjusted to zero, and the image was recorded and stored for background subtraction. The contrast of the target slots was then adjusted to 4 K, the image recorded, the background subtracted, and the gain of the camera-oscilloscope system calibrated. Figure 11, which does not reproduce well, shows the image of the target presented on the television screen during this calibration. Figure 12 shows an oscilloscope trace of one of the scan lines in the central part of the image shown in figure 11.

The camera was then directed toward the low-contrast target, and the latter was observed with zero voltage across the heater to record the background image. The background was observed to be unstable. The cause of the instability was traced to an image of the camera operator being inadvertently reflected into the camera’s field of view by the nonzero reflectance of the target. This points out the desirability of coating the top surface of the target with a thin, low-reflectance (high-emissivity) material such as gold black [3], not only to decrease the power required for a given contrast, but also to reduce the sensitivity to the background. To solve this problem for the existing devices, a black cloth was suspended around the target and camera, and a stable background was obtained. The latter was then recorded and stored for background subtraction.

**Figure 13:**
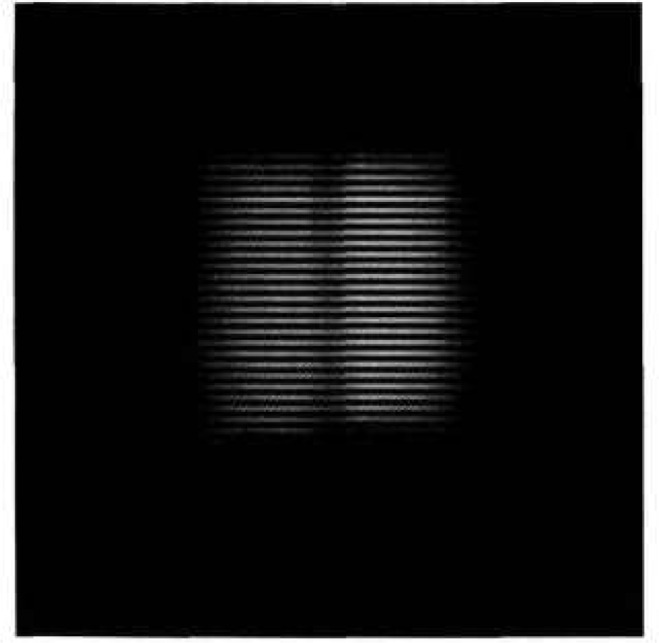
Television image of the new type of low-contrast thermal resolution test target obtained in the same way as that shown in figure 11.

**Figure 14:**
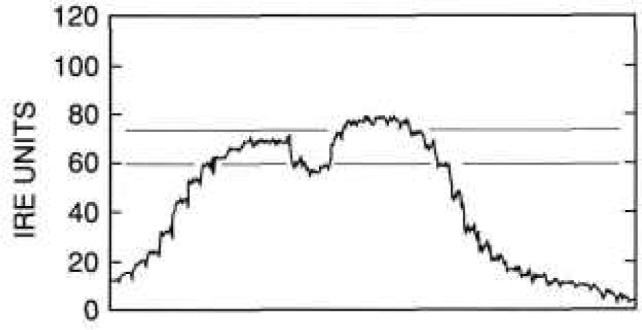
A scope trace of one of the scan lines in the image shown in figure 13. Nominal averages of the high- and low-temperature portions of the target are shown by the broken lines. The difference between the lines is about 13 IRE units.

The voltage across the heater was then increased until the contrast between the heated top surface of the target and the surrounding heat sink was about 4 K as measured on the calibrated oscilloscope, and the heater power was recorded. Figure 13, which also does not reproduce well, shows the image obtained under this condition. Figure 14 shows an oscilloscope trace of one of the TV raster-scan lines of the camera. This particular line is located near the central part of the image shown in figure 13.

The rounding of the temperature profile in figure 14 at the outside edges of the target was expected, as shown in the last section, because there was no heater. The rounding at the edges of the groove is partially an artifact caused by not assuring that the image of the groove exactly filled an integral number of pixels on the PtSi array in the camera. This was verified to be the case by translating the target perpendicular to the optical axis of the camera with a micrometer-driven translation stage. It was possible to make the pixel on either side of the groove assume any value between that in the center of the groove and the edge of the groove as the target was translated.

The cause of the temperature gradient across the target is not known. A variation in the thickness of either the epoxy between the heat sink and the target or of the inconel heater film on the top surface of the target could cause this. Since no precautions were taken to assure the uniformity of the epoxy joint between the target and heat sink, a variation in thermal resistance caused by a variation in epoxy thickness is the most likely cause for the temperature gradient observed with the camera. Since the thermal conductivity of the epoxy is about one-third that of fused silica, the thermal resistance of the 0.075-mm-thick layer of epoxy is about 33% of that of the 0.75-mm-thick fused silica layer. Therefore, a thickness gradient of the order of half the nominal thickness of the film would be needed to explain the observed temperature gradient. This is not unreasonably large considering that no attempt was made to obtain a uniform joint. Clearly, it would be desirable to devise a way to assure a uniform epoxy joint.

**Figure 15:**
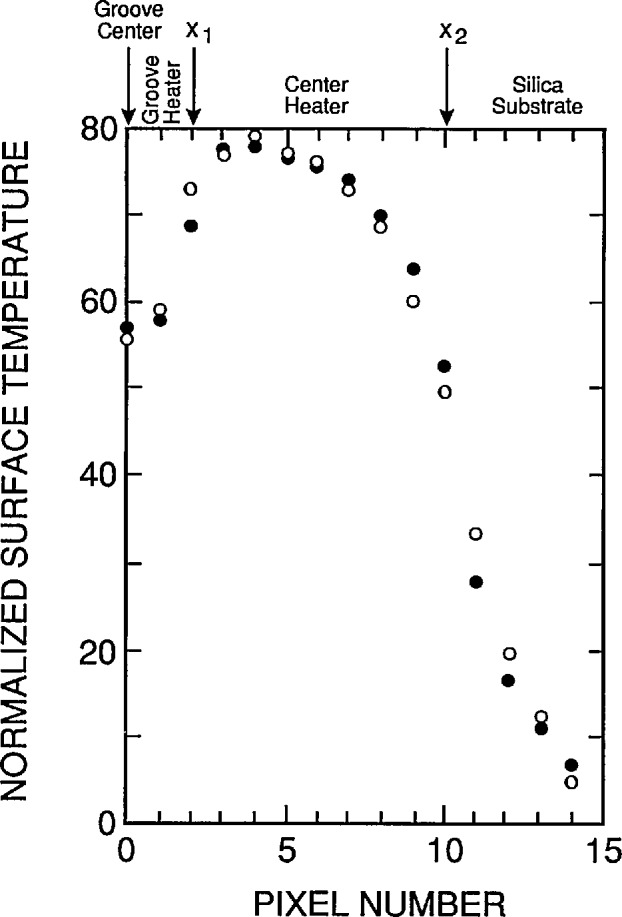
Comparison of two parameters fit (filled circles) of theoretical model to the experimental data (open circles) shown in figure 14. The data for the left- and right-hand sides of target were averaged to obtain the experimental data plotted here.

Figure 15 compares the theoretical and experimental temperature profiles for the device shown in figure 5. The experimental profile was obtained by reflecting the left-hand side of the oscilloscope trace of figure 14 about the center of the groove and averaging it with the right-hand side of the trace. The theoretical profile was obtained using the model described earlier. Since this model does not account for the epoxy joint between the target and the heat sink, the device was modeled as having a thickness of 1.00 mm instead of the measured thickness of 0.75 mm. This additional thickness gives the same thermal resistance between the top surface of the device and the heat sink as 0.75 mm of fused silica in thermal series with 0.075 mm of epoxy.

The model was fitted to the measured data by adjusting the groove depth until a good fit was obtained. The fit shown in figure 15 was obtained with a model groove depth of 0.20 mm, whereas the actual depth was 0.30 mm. The deeper groove on the actual device indicates that the actual contrast obtained is not as good as that predicted by the model. This could be associated with a nonuniform epoxy film or a small lateral thermal gradient existing in the heat sink under the groove. It might also be associated with a heat loss from the vertical groove wall of the actual device; a heat loss of this type would tend to reduce the contrast. Despite this unresolved problem with the fit of the calculated temperature profile from this very simple, ideal model to the data from the actual devices that we built, figure 15 makes it clear that all of the major effects are explained by this model. Other than unwanted temperature gradients, there should be little or no deviations from the model in optimized targets. The target that we designed was built before the model calculations were developed. Consequently, the target was used to test the model and provide insight for future target designs. Simple improvements in design (such as a groove depth more nearly equal to the target thickness and a heater covering the entire top surface of the target) should yield much more ideal devices.

## 5. Use of New Type Target with Conventional Target

The lower limit of thermal contrast available from a conventional target can be extended to thermal contrasts as low as those that can be resolved by any given camera with one of the new types of targets and an oscilloscope. The procedure is the following: 1) the camera/oscilloscope system is calibrated with the conventional target generating a reliably large thermal contrast, 2) the calibrated camera/oscilloscope system is used to calibrate the new type of target at the same order of thermal contrast, 3) the power in the new type of target is reduced to produce the desired thermal contrast, 4) the reduced thermal contrast is calculated using the formula presented below, and 5) the gain of the camera/oscilloscope system is increased and calibrated.

The formula for this type of calibration is

$$DT_{\text{low}}=\frac{\left(DT_{\text{std}}\right)\left(DR_{\text{cal}}\right)}{\left(DR_{\text{std}}\right)\left(P_{\text{cal}}\right)}P_{\text{low}},$$ (1)

where *DT*_low_ is the lower level of thermal contrast generated in the new type target, *P*_low_ is the power dissipated in the new type target to generate *DT*_low_, *DR*_cal_ is the difference between the in-groove and out-of-groove output from the camera/oscilloscope when the power *P*_cal_*>P*_low_ is dissipated in the new type target, *DT*_std_ is the thermal contrast from the conventional target, and *DR*_std_ is the difference between the in-slot and out-of-slot output from the camera/oscilloscope when the camera is pointed at the conventional target. It is important to notice that the gain of the camera/oscilloscope system must be the same for the traces from which *DR*_std_ and .*DR*_cal_ are read. Only after the calibration of the new type target at the higher power level can the camera gain be changed.

Figures 12 and 14 illustrate how the target shown in figure 5 could be used as a low-contrast target. The thermal contrast of the conventional target was set to 4 K for the camera/oscilloscope trace shown in figure 12. Therefore, *DT*_std_*/DR*_std_ is about 4 K per 75 IRE units^3^ =0.053 K per IRE unit. For the oscilloscope trace shown in figure 14, the voltage drop across and the current passing through the target heater were 8 V and 44.5 mA, respectively, and the thermal contrast between the groove and trace outside the groove is about 13 IRE units. Therefore, *DR*_cal_*/P*_cal_ = 15 IRE units per (8 V × 44.5 mA) = 42 IRE units per W. After the gain of the camera/oscilloscope system was increased and the voltage across the heater of the new type target decreased until the groove was just detectable in the image of the target, the voltage across and the current passing through the target heater were 3 V and 17.5 mA, respectively. (It is interesting that the groove could not be detected in the oscilloscope trace at this level, even though it could be detected in the thermal image.) Therefore, the minimum resolvable temperature difference is P_low_ = 52.5 mW, and *DT*_low_ = (0.053 K per IRE unit) × (42 IRE units per watt) × 0.0525 W = 0.12 K.

This minimum resolvable temperature difference of 120 mK, measurable with the PtSi camera reported above, is in good agreement with the value determined through very careful measurements against the conventional thermal resolution target. Why then is there a need for a new type of low-contrast target? The need is for the calibration of future cameras with better than 120-mK resolution. These experiments show that the new target is capable of calibrations to near 0 K. This is because the noise decreases linearly with the contrast or signal, and the signal-to-noise ratio remains constant allowing contrast calibrations to near 0 K. This would be impossible, or at least very difficult to do, with the conventional target.

## Figures and Tables

**Table 1:** Thermal conductivity of selected materials

Material	Thermal conductivity (W/cm K)
air	0.00024
epoxy	0.0043
silica	0.014
aluminum	2.1

**Table 2:** Electrical resistance of films

Material	Nominal thickness (nm)	Resistance (Ω/□)
aluminum	250 nm	0.14
inconel	10 nm	100.

## Footnotes

1 Certain commercial equipment, instruments or materials are identified in this paper to specify adequately the experimental procedure. Such identification does not imply recommendation or endorsement by the National Institute of Standards and Technology, nor does it imply that the materials or equipment identified are necessarily the best available for the purpose.

2 TURBO PASCAL is a registered trademark of Borland International, Scotts Valley, CA.

3 The vertical deflection of the oscilloscope trace, as seen in figures 12 and 14, are given in IRE units.

## 7. Appendix 2. Fabrication of the Test Targets

The targets were fabricated on 75-mm- (3-in-) diameter, 750-*µ*m-thick, fused silica wafers. In this fabrication process, grooves were first ultrasonically machined into the wafers at specified locations. Inconel-600 films^4^ 10 nm thick were sputter deposited on the wafers. Over the inconel films, pure aluminum films 250 nm thick were sputter-deposited. The target patterns were aligned to the ultrasonically machined grooves and patterned using photolithographic techniques. Only a small number of machined wafers were available which severely limited the amount of experimentation that could be done for process development. This number was further limited because the bottoms of the ultrasonically machined grooves were rough enough to prevent good electrical continuity in the 10-nm-thick inconel film deposited in them. Attempts to smooth these machined surfaces were made by dipping the wafers into 49% HF. After a few minutes etching, sharply outlined surface scratches appeared on the silica surfaces rendering some wafers useless for target fabrication.

The fabricated targets are shown schematically in the dimensioned drawing of figure 5. Side-by-side arrays of six of these targets were simultaneously fabricated on each wafer. The outer two targets on each side of the array, i.e., the targets closest to the periphery of the wafer, contained no grooves and served as control targets during fabrication.

Three different methods were used to fabricate these targets. One of these methods consisted of a lift-off technique which is described later. The other two consisted of differential etching which made use of the fact that the aluminum etchant etched inconel much slower than aluminum. This enabled the uppermost aluminum film to be patterned photolithographically and etched off of the inconel film. The primary differences in these two etching methods are shown in table A2.1. The resistances, in ohms, of the target heaters, fabricated by each method, are given in table A2.2. In this process, the first level was patterned into photoresist and consisted of the aluminum busses at the ends of the target grooves (shown in fig. 9). The aluminum was etched at 45 °C with a commercial aluminum etchant.^5^ The etching was stopped as soon as the aluminum film completely dissolved exposing the underlying inconel film. The resist was then stripped with acetone, and the second level, which consisted of the inconel heaters, was patterned into another photoresist film. This patterned photoresist film completely covered the aluminum busses protecting them from attack by the inconel etchant. The inconel was etched with Cyantek CR-9 Chromium Photomask Etchant,^6^ which is a concentrated perchloric acid-based etchant.

**Table A2.1:** Primary differences in the etching methods

Differential etch method 1	Differential etch method 2
Sputter Deposition vacuum broken between the depositions of inconel and aluminum	Sputter Deposition with aluminum deposited immediately over inconel without breaking vacuum
Photoresist—Shipley Micro-posit 1350J	Photoresist—Shipley Micro-posit 1470
Photoresist coated by conventional spinning and open hot plate baking	Photoresist coated by the flood-surface tension method and a “capped” hot plate bake

**Table A2.2:** Resistances^2^ of target heaters in ohms

	Target number on Wafer					
Method	l^b^	2	3	4	5	6^b^
Dif. etch method 1	138	33^c^	167	150	22^c^	136
Dif. etch method 2	163	257	244	293^d^	447^d^	157
Lift off	183	180	197	163	171	164

a These resistances are measured by touching the probes of an ohmmeter to the aluminum busses. The resistances of the busses and probe contacts are assumed to be negligible.

b Have no grooves and serve as control targets.

c These low values are attributed to the small strips of aluminum that were observed on the wafer surfaces along the grooves.

d Targets where the etching of the Shipley 314 developer was most severe.

Difficulty was encountered with photoresist building up on the sharp inside (the sides toward the wafer center) precipices of the grooves when it was spun on the wafer by a conventional method. The photoresist in this buildup could not be satisfactorily patterned. A “flood-surface-tension” method was developed for spin coating the photoresist that did not produce this unwanted photoresist buildup. In this method: 1) the wafer is mounted on the spinner chuck and completely flooded with photoresist; 2) immediately following the flooding, the wafer is spun at 300 to 400 rpm for approximately 1.5 s, and then ramped to 1500 rpm and spun for 5 s; 3) when the spinner stops, the wafer is allowed to set on the chuck for an additional 60 s; and then 4) prebaked, in a flat, horizontal position, on a hot plate at 95 °C for 3.5 min with an aluminum foil tent covering it and the immediate surrounding hot plate area. The purpose of the two short, slow spins is to remove excess photoresist and achieve a reasonably thin resist coating without producing either buildup in or significant solvent loss from the photoresist. The 60-s setting period allows the photoresist surface tension to planarize the resist and reduce any buildup. The pre-bake is done with the wafer flat to prevent uneven resist film formation, and the aluminum tent promotes better heating of the resist and improves its adhesion. It should be noted that the thermal conductivity of a 750-*µ*m- (30-mil-) thick fused silica wafer is substantially less than that of a silicon wafer and necessitated the use of the tent.

Exposures of uv were made at 180 to 220 mJ/cm^2^ using AR-chrome masks. Spray-puddle-developing was used with full-strength, Shipley 314 developer.^7^ Before exposures were made, the coated wafers were either allowed to set in the air for at least 1 h, or if the exposure had to be made sooner, the wafer was dipped in deionized water for 1 min and spun dry. The purpose of this step was to replace water in the photoresist film that was removed during the baking. This water is necessary for the resist photolysis to proceed.

Some problems were encountered with each of the differential etch methods. With method 1, thin lines of unetched aluminum were observed on the wafer surfaces adjacent to the sides of the grooves toward the wafer center. These lines are believed to be a result of resist buildup. With method 2, photoresist was observed on the groove walls after developing. The removal of this resist was attempted by re-exposing the groove walls and developing by puddling a drop of Shipley 314 developer in the grooves. The developer etched the aluminum where it was puddled which, in turn, caused the inconel to be etched thinner in these areas.

### 7.1 Lift-Off Method

Inconel was first sputter-deposited onto the wafer which was then conventionally spin-coated with Shipley 1350J and prebaked 3.5 min at 95 °C on a hot plate. A uv exposure of 220 mJ/cm^2^ was used to pattern a mask of the aluminum busses (the polarity of which was the reverse of the masks used in the differential etching methods) onto the wafer. After this exposure, the wafer was soaked in chlorobenzene for 5 min and spray-puddle-developed. A 250-nm aluminum film was evaporated, in a filament evaporator, onto the patterned wafer under a vacuum where P≤4 × l0^−5^ Torr. The distance between the filament and the wafer was about 50 cm. The filament was a trough filament into which a few pieces of braided tungsten wire were placed with aluminum pellets. When the aluminum melted, it wetted the tungsten wire which then gave even evaporation of the aluminum for the 250-nm-thick film. Lift-off of the aluminum film was then accomplished by soaking the wafer for 3 h in acetone with intermediate acetone flushes from a squeeze bottle to help dislodge the film. The aluminum in the corners of the grooves at the base of their vertical walls required “swabbing” with a sharp plastic point under acetone for complete removal.

The remaining inconel film was then patterned by coating the wafer with Shipley 1470 photoresist using the flood-surface-tension method, patterning the inconel mask with a 220 mJ/cm^2^ exposure, spray-puddle-developing with Shipley 314 developer, and etched with CR-9. The resistances of these inconel heaters are given in table A2.2.
